# Effects of sevoflurane exposure on apoptosis and cell cycle of peripheral blood lymphocytes, and immunologic function

**DOI:** 10.1186/s12871-021-01305-w

**Published:** 2021-03-20

**Authors:** Zhimin Ji, Wanjun Wu, Fan Zhou, Junfang Hu, Qiuping Xu, Weibin Yang, Xueyong Peng, Xinguo Wang, Cheng Zhang, Li Li

**Affiliations:** 1 Department of Anesthesiology, Puren Hospital Affiliated to Wuhan University of Science and Technology, Wuhan, China; 2 Department of Anesthesiology, the Affiliated Suzhou Science and Technology Town Hospital of Nanjing Medical University, Suzhou, China; 3 Department of Clinical Laboratory, Puren Hospital, Affiliated to Wuhan University of Science and Technology, Wuhan, China; 4 Department of Pharmacy, Puren Hospital, Affiliated to Wuhan University of Science and Technology, Wuhan, China; 5 Wuhan Municipal Health Commission, Wuhan, China; 6 Department of Occupational Health, Wuhan Prevention and Treatment Center for Occupational Disease, Wuhan, China; 7 Department of Pathology, Puren Hospital Affiliated to Wuhan University of Science and Technology, Wuhan, China

**Keywords:** Sevoflurane, Apoptosis, Cell cycle, Peripheral blood lymphocytes, Immunologic function

## Abstract

**Background:**

Waste anesthetic gases (WAGs) leaked from new-type halogenated inhalational anesthetics such as sevoflurane have been were reported to pose a risk for the health of operating room personnel. The effects of WAGs on peripheral blood lymphocytes, however, remain yet controversial. The present study was undertaken to examine the effects of occupational sevoflurane exposure on the peripheral blood lymphocytes of medical personnel who work in the operating room.

**Methods:**

A cohort of 56 medical residents were divided into exposed group (*n* = 28) and control group (non-exposed group) (*n* = 28). Gas chromatography was used to measure the concentration of sevoflurane in the medical resident’s breathing zone during surgeries under inhalation anesthesia in the exposure group. The gas collection lasted an hour. Peripheral blood lymphocytes were isolated from venous blood, and then apoptosis and cell cycle were analyzed by flow cytometry. EDTA-anticoagulated whole blood was harvested to analyze the lymphocyte subsets by flow cytometry. Immunoglobulins (IgA, IgM, IgG) were quantified by immunoturbidimetry.

**Results:**

The average concentration of sevoflurane in the exposed group was 1.03 ppm with a range from 0.03 ppm to 2.24 ppm. No significant effects were found on the apoptosis rates or cell cycles of peripheral blood lymphocytes in the exposed group relative to the control group (*P* > 0.05). Similarly, there were no significant differences in the lymphocyte subsets or the levels of immunoglobulins (IgA, IgM, IgG) between the two groups (*P* > 0.05).

**Conclusions:**

Occupational exposure to low-level sevoflurane has no significant effect on the peripheral blood lymphocytes of operating room staff, but this conclusion needs to be confirmed by multicenter and long-term follow-up studies with large samples.

**Trial registration number and date of registration:**

ChiCTR2000040772, December 9, 2020 (Retrospective registration).

## Background

With the advantage of low solubility in blood and high controllability, inhalation anesthetics are widely used in the operating room (OR), dental clinic, delivery room, MRI room and intensive care unit [1, 2]. Despite many improvements in the anesthesia equipment and the production of safer anesthetic agents during the past decades, inhalation anesthetics, including halothane, isoflurane, sevoflurane, desflurane, and nitrous oxide (N_2_O), inevitably cause waste anesthetic gases (WAGs) in the workplace [3, 4]. Furthermore, chronic occupational exposure to the volatile anaesthetics has been reported to negatively impact the health of hospital personnel since these agents were introduced into clinical use in the 1990 s [1, 5, 6]. Some researchers believe, however, that there are no statistically sound studies identifying the concentrations of anesthetic gases that would exert harmful effects [7–9]. Moreover, the effects of the inhalational anesthetics on lymphocytes apoptosis remain controversial. Matsuoka et al. found that sevoflurane induced apoptosis in peripheral lymphocytes in dose-dependent and time-dependent manners *in vitro *[10]. Loop et al. also documented that sevoflurane induced T cells apoptosis *in vitro *[11]. On the contrary, Aun et al. failed to reveal significant differences in the percentages of viable and early apoptotic cells detected by flow cytometry between medical residents with and without brief occupational exposure [12]. Thus, the present cohort study was undertaken to ascertain whether occupational exposure to sevoflurane has harmful effects on the peripheral blood lymphocytes of medical personnel exposed to inhalational anesthetics *in vivo*.

## Methods

### Study design and occupational exposure

This study was approved by the Human Medical Research Ethics Review Board, Puren Hospital (prll2018001) prior to its initiation. All subjects who participated in this study provided written informed consent. This study was conducted in accordance with the stringent ethical requirements for research on human subjects. All participants answered a standardized and detailed questionnaire that included demographic data, medical history, lifestyle and anesthetic exposure before each blood sample was collected. To avoid the effects of possible confounding factors, subjects were excluded from the study if they were pregnant women, had any chronic infectious or inflammatory disease, were using illicit substances, medications, vitamins, and/or antioxidant supplements, had recently been exposed to radiation (within 6 month), or had medical or family history of blood diseases. The exposed group consisted of 28 anesthetists who were mainly exposed to sevoflurane for at least 24 months. The control group comprised 28 residents from internal medicine who were not exposed to WAGs or other pollutants. The exposed group was age- and sex-matched with the unexposed group. The biological sampling was performed from 2019 to 2020.

### Measurement of WAGs in the breathing zone of medical residents during surgeries

The Puren hospital maintains 13 ORs with an average area of 25 square meters each. The ORs do not have scavenging systems, but instead maintain vertical laminar flow conditions. All the medical residents from the exposed group worked in the 13 ORs in the hospital. All the WAGs were collected from the breathing zone of medical residents during the conduct of surgical inhalation anesthesia in all the ORs. The gas collection lasted an hour. The measurements were performed by using a GilAir-5 sampler (Sensidyne, USA) and Agilent 7890BGas Chromatograph System (Agilent Technologies, USA) according to the instruction of the manufacturer. The average of the gas concentration of sevoflurane was calculated and shown in ppm. Measurement requirement was in accordance with procedures approved by the National Institute for Occupational Safety and Health (NIOSH) [13].

### Peripheral blood lymphocyte preparation

EDTA anticoagulated venous blood samples were collected from median cubital vein of all participants. Peripheral blood lymphocytes were separated within subsequent 10–20 min (min) with a standard method by centrifugation over lymphocyte separation medium at 400×g for 20 min (lymphoprep™, STEMCELL technologies, Canada). The cells were washed with phosphate-buffered saline (PBS) (250×g, 10 min). Subsequently, 5 × 10^5^/ml peripheral blood mononuclear cells (PBMCs) suspended in the PBS were used in all the experiments.

### Assessments of apoptosis

The percentages of viable (annexin-/propidium iodide [PI]-) or early apoptotic (annexin+/PI-) cells were quantified by using annexin V-fluorescein isothiocyanate (FITC) staining, which was used to detect phosphatidylserine that is externalized in the early phases of apoptosis. Annexin V is an important marker of early apoptosis in which changes in externalized phosphatidylserine levels occur prior to DNA fragmentation [14]. We used the Annexin V-FITC Apoptosis Analysis Kit according to the manufacturer’s instructions (Tianjin Sungene Biotech Co., Ltd., China). Mononuclear cells (1 × 10^5^) were labeled, incubated in the dark for 15 min and immediately sorted by flowcytometry (BD FACSAria^TM^ш, USA). Marked annexin V-FITC staining (green) was analyzed using the FL-1channel, while PI staining (red) was assessed using the FL-2 channel. The data were analyzed by using the FlowJo software on a BD FACS Aria™ cytometer (BD BioSciences, USA).

### Cell cycle analysis

Cell cycle was examined by flow cytometry according to a previous report [15]. Briefly, cells (1 × 10^6^) were harvested and washed with 10 ml PBS by centrifugation for 5 min at 300×g, and then re-suspended in 0.5 ml PBS. They were fixed by adding 4.5 ml pre-chilled 70 % ethanol while vortexing. After incubation for 2 h at 4 °C, residual ethanol was eliminated by centrifugation for 5 min at 300×g. Supernatants were removed and discarded. Cells were washed with 5 ml FACS buffer twice by centrifugation for 5 min at 200×g. Cells were stained using 0.5 ml propidium iodide staining solution and kept in dark. Afterwards, they were incubated for 20 min at room temperature and the fluorescence analyzed using the ModFit LT software on BD FACSCalibur cytometry (BD, BioSciences USA).

### Analysis of subpopulations of lymphocyte by flow cytometry

EDTA-anticoagulated whole blood were freshly collected from median cubital vein and processed within 2 h by ten-color flow cytometry (BD FACSCanto™, USA) to analyze the lymphocyte subsets as previously described [16, 17]. The lymphocyte subsets were identified using the following monoclonal antibodies: anti-CD3-APC-H7, anti-CD8-PerCP-cy5.5, anti-CD4-BV605, anti-CD25-PE, anti-CD56-BV510, anti-CD19-APC, and anti-γδ-BV421 from BD Biosciences; anti-CD28-PE-CY7 and anti-CD127-FITC from Biolegend. The cell suspension was incubated at room temperature in the dark for 30 min. Red blood cells were removed using 500 µl of lysis buffer at room temperature in the dark for 10 min. Finally, the cells were analyzed by using FACS Canto flow cytometry and FlowJo software (BD BioSciences, USA).

### Immunoglobulin quantification by immunoturbidimetry

 Blood of the median cubital vein was collected into separate gel coagulant tub and the content of immunoglobulins (IgA, IgG, IgM) in the serum was detected by Immunoturbidimetric Assay according to the instructions of immunoglobulin assay kit (Shanghai Fosun Long March Medical Science co., LTD) on an automatic biochemical analyzer (beckmancoulterAU5800, USA).

### Statistical analysis

Data were analyzed using an SPSS software version 25. The sample size for this study was calculated based on a pilot study, with a test power of 80 % and level of 5 % of significance (mean expected differences of 7.02 and 7.85 standard deviations between two groups), and was determined to be 52 subjects. By adding a 10 % sample shedding rate, finally, the sample size was 56. Independent student’s *t*-test was used for comparing the mean of quantitative variables, and chi-square for comparing the mean of qualitative variables. *P*-values less than 0.05 were considered to be significant.

## Results

### Demographic characteristics of the study populations

The demographic characteristics of the exposed and the control groups are shown in Table 1. No statistically significant differences were noted between the two groups in these variables (age and sex) (*P* > 0.05).

**Table 1:** The characteristics of the study populations

	Control group (n=28)	Exposed group (n=28)	*P* value
Age(years)			0.18^*^
Mean±SD	34.04±6.03	32.14±4.34	
Range	25-48	27-46	
Sex (male/female)			
male	17	15	0.59^#^
Female	11	13	
Duration of exposure(years)			
Range		2-20	

^*^Independent sample t test

^#^Chi-square test

### Concentration of sevoflurane in exposed group is lower than the standard of NIOSH

After analyzing the concentration of sevoflurane collected from the breathing zone of all residents in the exposed group, we found that the average concentration of sevoflurane was 1.03 ppm with a range from 0.03 ppm to 2.24 ppm. The value was lower than the limit recommended by NIOSH.

### Sevoflurane exposure has no significant effect on apoptosis and cell cycle of peripheral blood lymphocytes

**Fig. 1:**
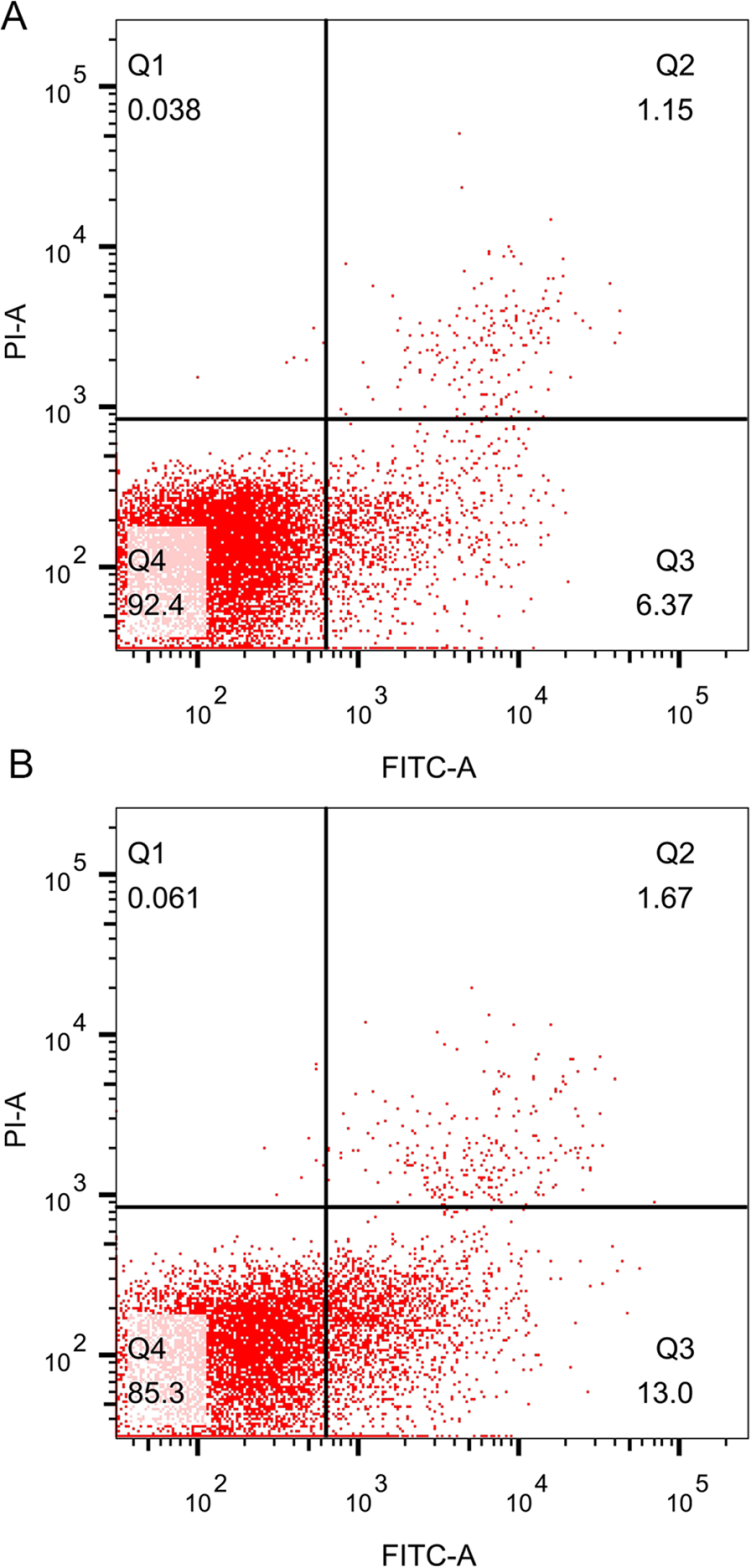
Apoptosis of peripheral blood lymphocyte analyzed by flow cytometry. **a** is representative picture of control group. **b** is representative picture of exposed group

**Fig. 2:**
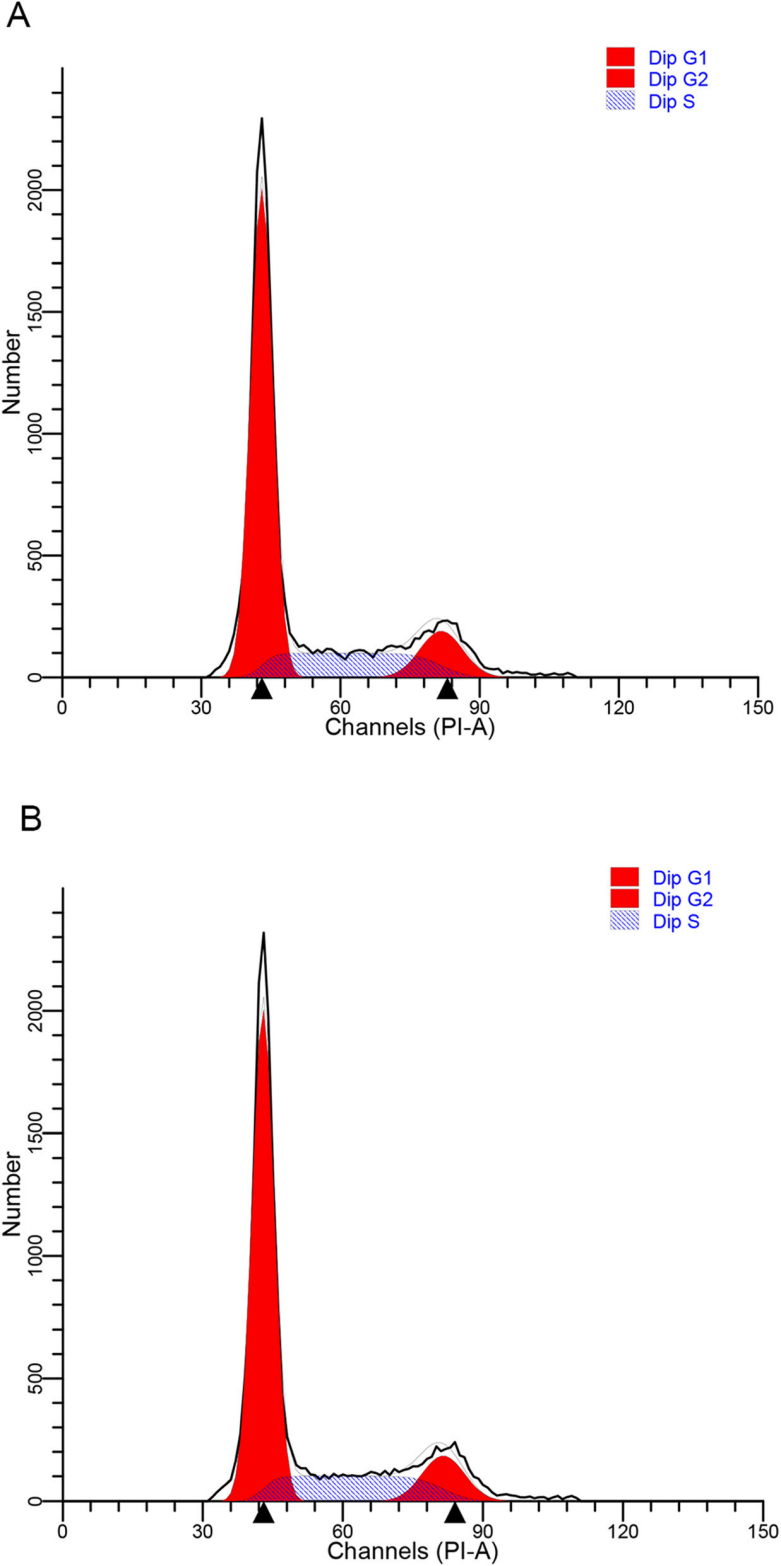
Cell cycle of peripheral blood lymphocyte analyzed by flow cytometry. **a** is representative picture of control group. **b** is representative picture of exposed group

**Fig. 3:**
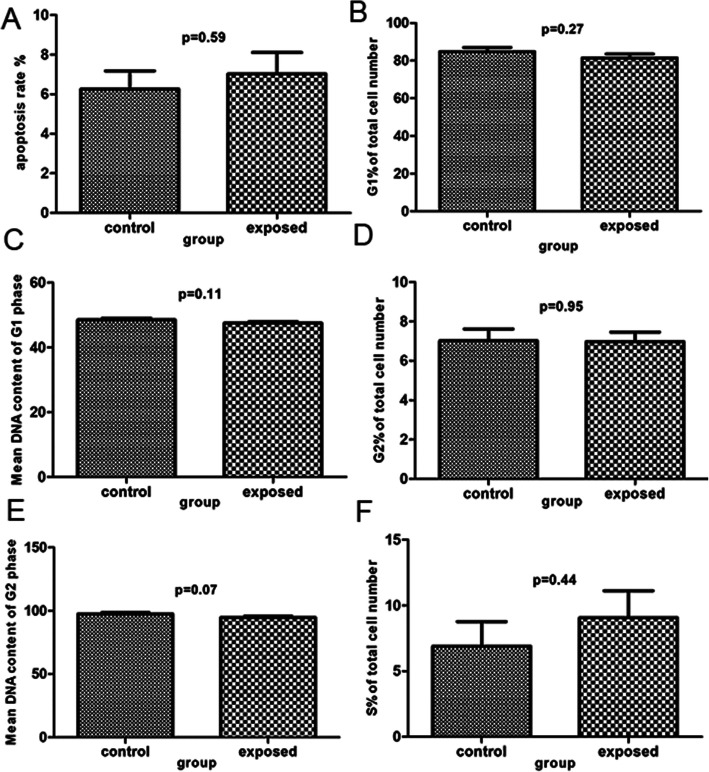
Cartogram of apoptosis rate and cell cycle. **a** is comparation of apoptosis rate of control group and exposed group. **b**, **d** and **f** represent percentage of G1 phase, G2 phase and S phase of cell cycle. **c** and **e** display mean DNA content of G1 phase and G2 phase respectively

As shown in Table 2; Figs. 1, 2 and 3, we found that there were no statistically significant differences in the apoptosis rates or cell cycles of the peripheral blood lymphocytes between control group and exposed group (*P* > 0.05). In addition, sevoflurane exposure had no significant effects on cells at G1 phase, G2 phase or S phase in the exposed group compared with the control group (*P* > 0.05). Although the proportion of S-phase cells and the apoptosis rate were slightly higher in the exposed group than in the control group, the differences were not statistically significant (*P* > 0.05).

**Table 2:** Comparison of apoptosis rate and cell cycle between control group and exposed group

Cell cycle	group	N	mean	SD	t	*P* value
G1% of total cell number	control	28	84.79	11.36	1.11	0.27
	exposed	28	81.49	11.0		
Mean DNA content of G1 phase	control	28	48.54	2.46	1.62	0.11
	exposed	28	47.50	2.33		
G2% of total cell number	control	28	7.02	3.17	0.06	0.95
	exposed	28	6.98	2.56		
Mean DNA content of G2 phase	control	28	97.41	5.98	1.83	0.07
	exposed	28	94.60	5.47		
S% of total cell number	control	28	6.89	9.92	-0.79	0.44
	exposed	28	9.07	10.83		
apoptosis rate	control	28	6.26	4.87	-0.542	0.59
	exposed	28	7.03	5.73

### Sevoflurane exposure has no significant impacts on subpopulations of lymphocytes

**Fig. 4:**
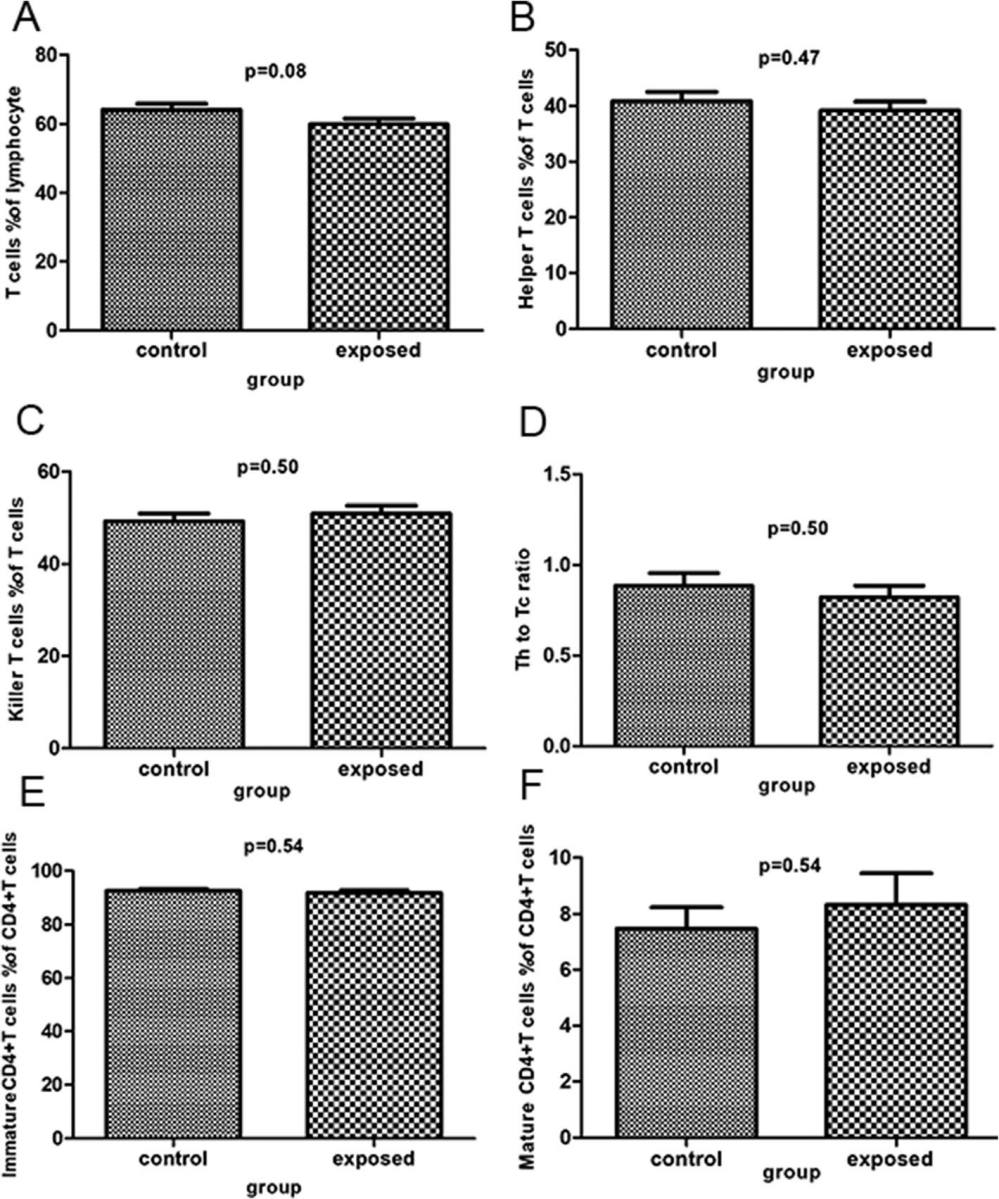
Analysis of subpopulation of lymphocyte by flow cytometry. **a**-**f** reveal various subpopulation of lymphocyte as follows: T cells % of lymphocyte, Helper T cells % of lymphocyte, Killer T cells % of T cells, Th to Tc ratio, ImmatureCD4+T cells % of CD4+T cells, Mature CD4+T cells % of CD4+T cells

**Fig. 5:**
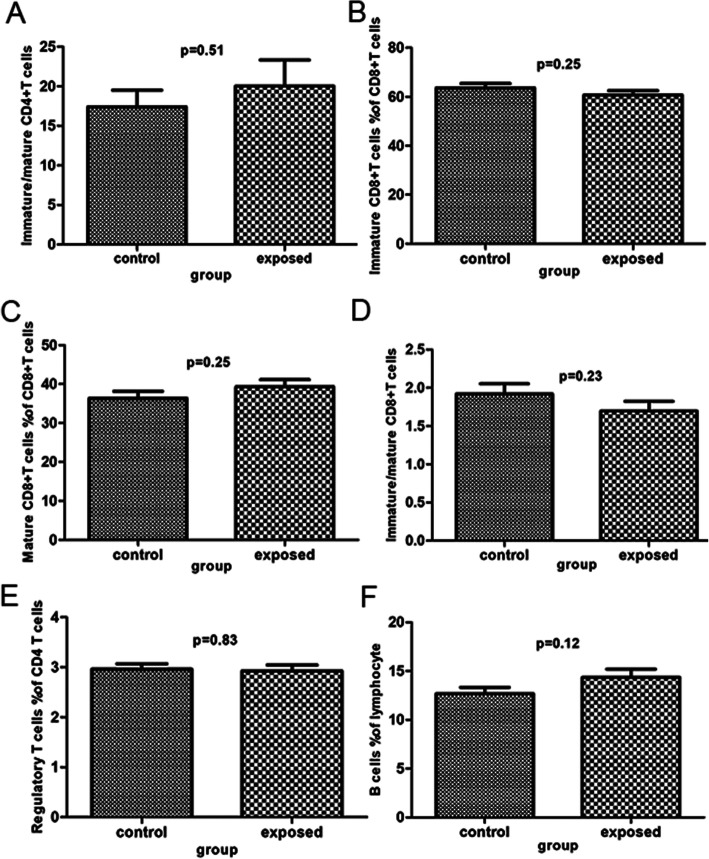
Analysis of subpopulation of lymphocyte by Flow cytometry. **a**-**f** reveal various subpopulation of lymphocyte as follows: Immature/mature CD4+T cells, Immature CD8+T cells % of CD8+T cells, Mature CD8+T cells % of CD8+T cells, Immature/mature CD8+T cells, Regulatory T cells % of CD4 T cells, B cells % of lymphocyte

**Fig. 6:**
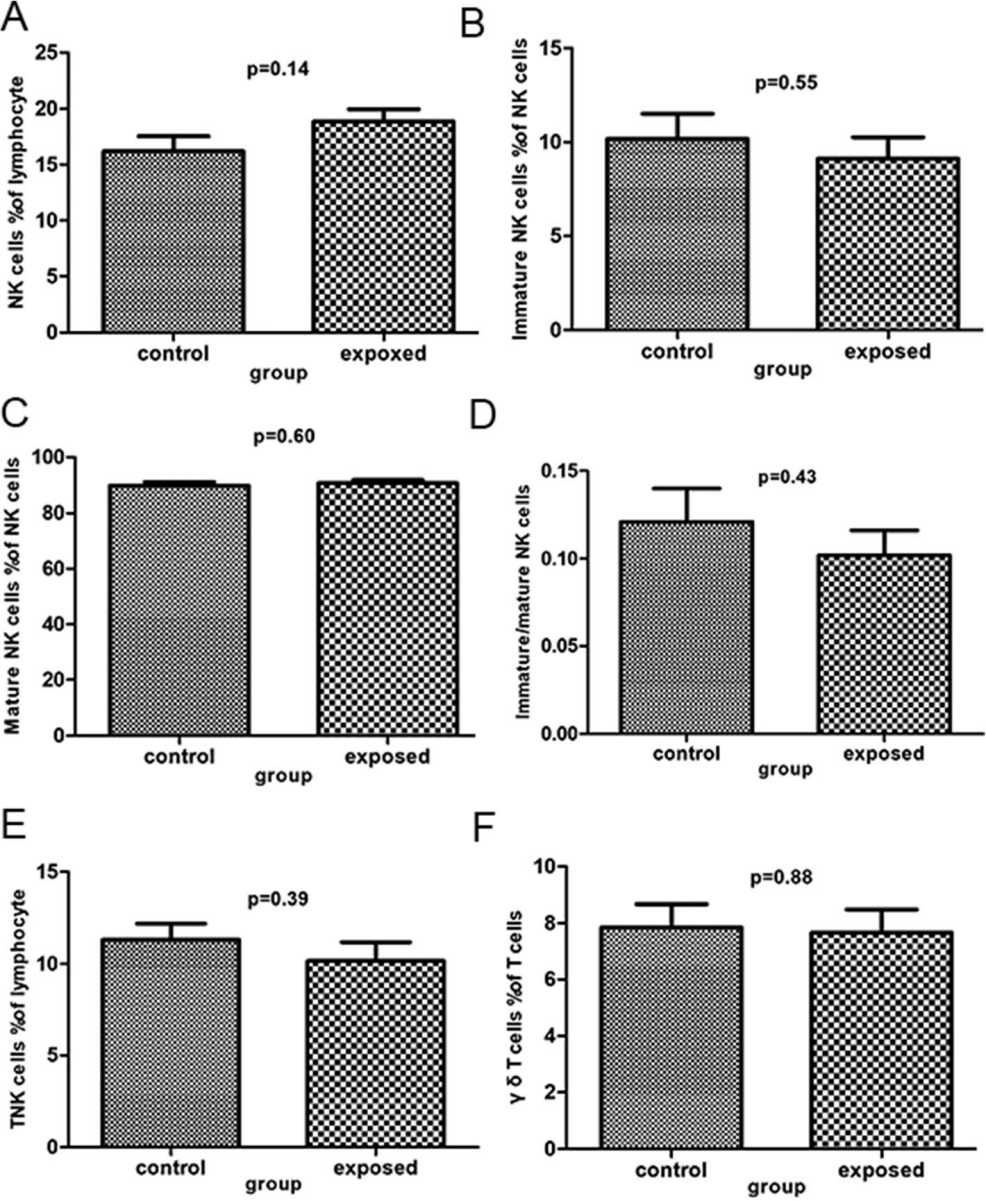
Analysis of subpopulation of lymphocyte by Flow cytometry. **a**-**f** show various subpopulation of lymphocyte as follows: NK cells% of lymphocyte, Immature NK cells % of NK cells, Mature NK cells % of NK cells, Immature/mature NK cells, TNK cells % of lymphocyte, γδT cells % of T cells

There were no statistically significant differences in all subpopulations of lymphocytes between the exposed group and the control group (*P* > 0.05, Table 3; Figs. 4, 5 and 6). Specifically, the percentages of helper T cells, killer T cells, Th to Tc ratio, immature CD4 + T cells, regulatory T cells, mature NK cells and γδT cells in the exposed group were not significantly different from those in the control group. There was a trend toward slightly lower percentages of mature CD4 + T cells, mature CD8 + T cells, NK cells, immature/mature CD4 + T cells and B cells in the control group compared to the exposed group, but the differences were not statistically significant. In addition, the percentages of T cells, immature CD8 + T cells, immature/mature CD8 + T cells, immature NK cells, immature/mature NK cells and TNK cells tended to increase in the control group relative to the exposed group (*P* > 0.05).

**Table 3:** Comparison subpopulation of Lymphocyte between control group and exposed group

subpopulation of lymphocyte	group	N	mean	SD	t	*P* value
T cells% of lymphocyte	control	28	64.18	8.76	1.81	0.08
	exposed	28	59.91	8.86		
Helper T cells% of T cells	control	28	40.83	8.75	0.72	0.47
	exposed	28	39.17	8.43		
Killer T cells% of T cells	control	28	49.29	8.93	-0.67	0.50
	exposed	28	50.92	9.18		
Th to Tc ratio	control	28	0.89	0.37	0.68	0.50
	exposed	28	0.82	0.33		
ImmatureCD4+T cells% of CD4+T cells	control	28	92.53	4.06	0.62	0.54
	exposed	28	91.70	5.96		
Mature CD4+T cells% of CD4+T cells	control	28	7.47	4.06	-0.62	0.54
	exposed	28	8.32	5.96		
Immature/mature CD4+T cells	control	28	17.41	11.08	-0.67	0.51
	exposed	28	20.03	17.43		
Immature CD8+T cells% of CD8+T cells	control	28	63.64	9.49	1.16	0.25
	exposed	28	60.69	9.64		
Mature CD8+T cells% of CD8+T cells	control	28	36.35	9.49	-1.16	0.25
	exposed	28	39.31	9.64		
Immature/mature CD8+T cells	control	28	1.92	0.70	1.22	0.23
	exposed	28	1.70	0.67		
Regulatory T cells% of CD4 T cells	control	28	2.96	0.58	0.21	0.83
	exposed	28	2.93	0.63		
B cells% of lymphocyte	control	28	12.68	3.42	-1.59	0.12
	exposed	28	14.36	4.41		
NK cells% of lymphocyte	control	28	16.19	7.25	-1.52	0.14
	exposed	28	18.86	5.87		
Immature NK cells% of NK cells↓	control	28	10.18	7.08	0.60	0.55
	exposed	28	9.14	5.98		
Mature NK cells% of NK cells↓	control	28	89.81	7.08	-0.53	0.60
	exposed	28	90.74	5.92		
Immature/mature NK cells	control	28	0.12	0.10	0.80	0.43
	exposed	28	0.10	0.08		
TNK cells% of lymphocyte	control	28	11.32	4.63	0.86	0.39
	exposed	28	10.15	5.44		
γδ T cells% of T cells	control	28	7.85	4.33	0.16	0.88
	exposed	28	7.67	4.31

### Sevoflurane exposure has no influence on the levels of immunoglobulins

**Fig. 7:**
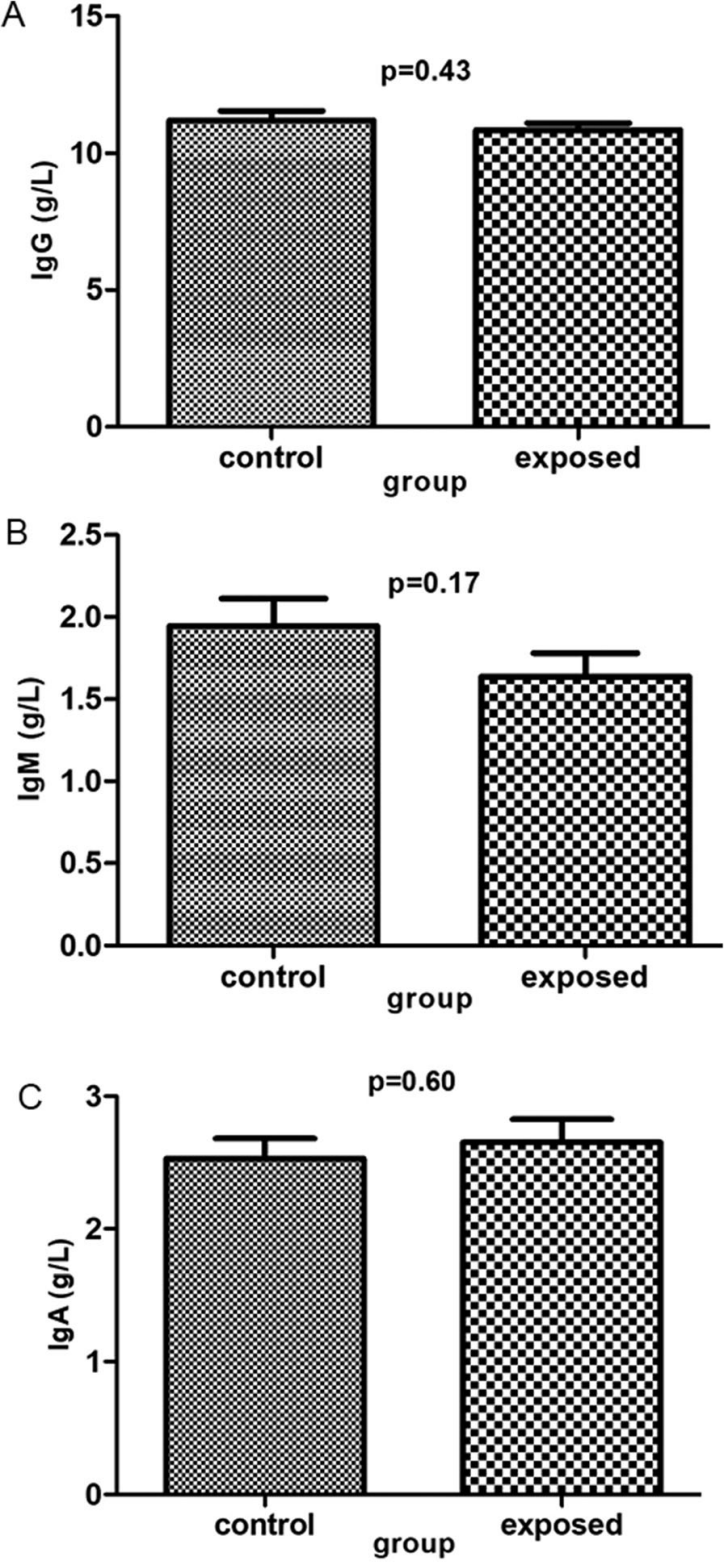
statistical graph of immunoglobulin quantification by immunoturbidimetry. **a**, **b** and **c** represent quantification of Ig G, IgM and IgA respectively

Immunoglobulins, including IgA, IgM, and IgG, were not significantly different in the exposed group from those in the control group (*P* > 0.05 Table 4; Fig. 7).

**Table 4:** Comparison of Immunoglobulin quantification between control group and exposure group

Immunoglobulin quantification(g/l)	group	N	mean	SD	t	*P* value
IgG	control	28	11.20	1.85	0.80	0.43
	exposed	28	10.85	1.37		
IgM	control	28	1.94	0.88	1.41	0.17
	exposed	28	1.64	0.75		
IgA	control	28	2.53	0.80	-0.53	0.60
	exposed	28	2.65	0.92

## Discussion

Our study demonstrated that the concentration of sevoflurane in the breathing zone of medical residents working in the OR was lower than the standard of NIOSH. In addition, chronic sevoflurane exposure had no significant effects on the apoptosis rates or cell cycles of peripheral blood lymphocytes in exposed medical staff. Finally, there were no significant differences in the subpopulations of lymphocytes or the levels of immunoglobulins (IgA, IgM, IgG) between the exposed group and controls.

Consistently, a recent study by Aun et al. did not found significant differences in the percentages of viable or early apoptotic cells detected by flow cytometry among medical residents with brief occupational exposure *in vivo.* Their study was performed before the medical residency program (no exposure; the physicians served as their own controls) and again after 1/2 year and 1 year of exposure [12]. Though previous studies found that sevoflurane induced apoptosis in peripheral lymphocytes *in vitro *[10, 11], we believe that the findings from experiments *in vitro* cannot be directly applied to actual conditions *in vivo*. The reason for this inconsistency may be speculated as follows: (1) such low concentration of sevoflurane is not enough to cause remarkable influences on peripheral blood lymphocytes. (2) Some regulatory mechanisms *in vivo* may correct the effects of sevoflurane exposure on peripheral blood lymphocytes. (3) Regeneration of peripheral blood lymphocytes alleviates the degree of cell injury from sevoflurane. Indeed, based on prior studies, there is not sound evidence that trace concentrations of anesthetic gases exert harmful effects [7]. Only high concentration levels of anesthetic agents and long-time exposure have been proven to result in significant histotoxicity [7]. Byhahn believe, under modern air conditioning, personnel’s occupational exposure is low, and inhalational anesthesia is safe from the standpoint of modern workplace laws and health care regulations [18]. Our study further supports the conclusions mentioned above by analyzing the actual effects of sevoflurane on apoptosis and cell cycle of peripheral blood lymphocytes, and immunologic function *in vivo*; and it was revealed that exposure to low level of sevoflurane has no significant harmful effects on peripheral blood lymphocytes of OR staff.

Nevertheless, there are some limitations in this study. First of all, all the subjects were limited to one hospital, and this environment may not be generalizable to other institutions. Secondly, there is a lack of long-term observation of the dynamic changes of the variables in the subjects. Multicenter and long-term follow-up studies with large samples are warranted to further confirm our results. Research about the effect of exposure to WAGs on cognitive function in addition to immune function is also worth investigation in the future.

## Conclusions

In the modern laminar-flow OR, medical personnel’s occupational exposure to sevoflurane does not exceed standard limits. Chronic occupational exposure to sevoflurane (less than limit of exposure) was found to have no significant harmful effect on the peripheral lymphocytes of OR staff.

## Data Availability

The datasets used and analyzed during the current study are available from the corresponding author on reasonable request.

## References

[1] Souza KM, Braz LG, Nogueira FR, Souza MB, Bincoleto LF, Aun AG et al, et al. Occupational exposure to anesthetics leads to genomic instability, cytotoxicity and proliferative changes. Mutation Res. 2016;791–792. 10.1016/j.mrfmmm.2016.09.002 27639372

[2] Deng HB, Li FX, Cai YH, Xu SY (2018). Waste anesthetic gas exposure and strategies for solution. J Anesth.

[3] Vodicka P, Musak L, Fiorito G, Vymetalkova V, Vodickova L, Naccarati A, et al.DNA and chromosomal damage in medical workers exposed to anaesthetic gases assessed by the lymphocyte cytokinesis-block micronucleus (CBMN) assay. A critical review. Mutation Res. 2016;770:26–34. 10.1016/j.mrrev.2016.04.003 27894688

[4] Lucio LMC, Braz MG, do Nascimento Junior P, Braz JRC, Braz LG (2018). [Occupational hazards, DNA damage, and oxidative stress on exposure to waste anesthetic gases]. Rev Bras Aanestesiol.

[5] Kunze N, Weigel C, Vautz W, Schwerdtfeger K, Junger M, Quintel M et al, et al. Multi-capillary column-ion mobility spectrometry (MCC-IMS) as a new method for the quantification of occupational exposure to sevoflurane in anaesthesia workplaces: an observational feasibility study. J Occup Med Toxicol. 2015;10:1–9. 10.1186/s12995-015-0056-7 PMC4379543 25829942

[6] Braz MG, Souza KM, Lucio LMC, Di Renzo GCC, Feliciano LM, Marcondes JPC et al, et al. Detrimental effects detected in exfoliated buccal cells from anesthesiology medical residents occupationally exposed to inhalation anesthetics: An observational study. Mutation Res Genet Toxicol Environ Mutagen. 2018;832–833. 10.1016/j.mrgentox.2018.07.001 30057022

[7] Ferstandig LL (1978). Trace concentrations of anesthetic gases: a critical review of their disease potential. Anesthes Anal.

[8] Szyfter K, Stachecki I, Kostrzewska-Poczekaj M, Szaumkessel M, Szyfter-Harris J, Sobczynski P (2016). Exposure to volatile anaesthetics is not followed by a massive induction of single-strand DNA breaks in operation theatre personnel. J Appl Genet.

[9] Amiri F, Neghab M, Shouroki FK, Yousefinejad S, Hassanzadeh J (2018). Early, Subclinical Hematological Changes Associated with Occupational Exposure to High Levels of Nitrous Oxide. Toxics.

[10] Matsuoka H, Kurosawa S, Horinouchi T, Kato M, Hashimoto Y (2001). Inhalation anesthetics induce apoptosis in normal peripheral lymphocytes in vitro. Anesthesiology.

[11] Loop T, Dovi-Akue D, Frick M, Roesslein M, Egger L, Humar M (2005). Volatile anesthetics induce caspase-dependent, mitochondria-mediated apoptosis in human T lymphocytes in vitro. Anesthesiology.

[12] Aun AG, Golim MA, Nogueira FR, Souza KM, Arruda NM, Braz JRC et al, et al. Monitoring early cell damage in physicians who are occupationally exposed to inhalational anesthetics. Mutation Res. 2018;812. 10.1016/j.mrfmmm.2018.10.002 30388507

[13] National Institute of Occupational Safety and Health (NIOSH):NIOSH Pocket Guide to Chemical Hazards. Washington, DC, U.S.; 1994.

[14] van Engeland M, Nieland LJ, Ramaekers FC, Schutte B, Reutelingsperger CP (1998). Annexin V-affinity assay: a review on an apoptosis detection system based on phosphatidylserine exposure. Cytometry.

[15] Kim KH, Sederstrom JM, et al. Assaying Cell Cycle Status Using Flow Cytometry. Curr Prot Mol Biol. 2015;111. 10.1002/0471142727.mb2806s111 PMC4516267 26131851

[16] Kotfis K, Biernawska J, Zegan-Baranska M, Zukowski M (2015). Peripheral Blood Lymphocyte Subsets (CD4+, CD8 + T Cells, NK Cells) in Patients with Cardiovascular and Neurological Complications after Carotid Endarterectomy. Int J Mol Sci.

[17] Lubbers J, van Beers-Tas MH, Vosslamber S, Turk SA, de Ridder S, Mantel E (2016). Changes in peripheral blood lymphocyte subsets during arthritis development in arthralgia patients. Arthritis Res Ther.

[18] Byhahn C, Heller K, Lischke V, Westphal K (2001). Surgeon’s occupational exposure to nitrous oxide and sevoflurane during pediatric surgery. World J Surg.

